# Embodied neuromorphic intelligence

**DOI:** 10.1038/s41467-022-28487-2

**Published:** 2022-02-23

**Authors:** Chiara Bartolozzi, Giacomo Indiveri, Elisa Donati

**Affiliations:** 1 Event-Driven Perception for Robotics, Istituto Italiano di Tecnologia, via San Quirico 19D, 16163 Genova, Italy; 2 Institute of Neuroinformatics, University of Zurich and ETH Zurich, Winterthurerstr. 190, 8057 Zurich, Switzerland

**Keywords:** Electrical and electronic engineering, Applied mathematics, Computational science, Computer science, Electronic devices

## Abstract

The design of robots that interact autonomously with the environment and exhibit complex behaviours is an open challenge that can benefit from understanding what makes living beings fit to act in the world. Neuromorphic engineering studies neural computational principles to develop technologies that can provide a computing substrate for building compact and low-power processing systems. We discuss why endowing robots with neuromorphic technologies – from perception to motor control – represents a promising approach for the creation of robots which can seamlessly integrate in society. We present initial attempts in this direction, highlight open challenges, and propose actions required to overcome current limitations.

## Abstract (web summary)

A grand challenge in robotics is realising intelligent agents capable of autonomous interaction with the environment. In this Perspective, the authors discuss the potential, challenges and future direction of research aimed at demonstrating embodied intelligent robotics via neuromorphic technology.

## Opportunities and challenges

Neuromorphic circuits and sensorimotor architectures represent a key enabling technology for the development of a unique generation of autonomous agents endowed with embodied neuromorphic intelligence. We define intelligence as the ability to efficiently interact with the environment, to plan adequate behaviour based on the correct interpretation of sensory signals and internal states, for accomplishing its goals, to learn and predict the effects of its actions, and to continuously adapt to changes in unconstrained scenarios. Ultimately, embodied intelligence allows the robot to interact swiftly with the environment in a wide range of conditions and tasks^1^. Doing this “efficiently” means performing robust processing of information with minimal use of resources such as power, memory and area, while coping with noise, variability, and uncertainty. These requirements entail finding solutions which improve performance and increase robustness in a way that is different from the standard engineering approach of adding general purpose computing resources, redundancy, and control structures in the system.

Current progress in both machine learning and computational neuroscience is producing impressive results in Artificial Intelligence (AI)^2–4^. However, conventional computing and robotic technologies are still far from performing as well as humans or other animals in tasks that require embodied intelligence^1,5^. Examples are spatial perception tasks for making long-term navigation plans, coupled with fine motor control tasks that require fast reaction times, and adaptation to external conditions. Within this context, a core requirement for producing intelligent behaviour is the need to process data on multiple timescales. This multi-scale approach is needed to support immediate perception analysis, hierarchical information extraction and memorisation of temporally structured data for life-long learning, adaptation and memory reorganisation. While conventional computing can implement processes on different timescales by means of high-precision (e.g. 32-bit floating point) numerical parameters and long-term storage of data in external memory banks, this results in power consumption figures and area/volume requirements of the corresponding computational substrate that are vastly worse than those of biological neural networks^6^.

The neuromorphic engineering approach employs mixed-signal analogue/digital hardware that supports the implementation of neural computational primitives inspired by biological intelligence that are radically different from those used in classical von Neumann architectures^7^. This approach provides energy-efficient and compact solutions that can support the implementation of intelligence and its embodiment on robotic platforms^8^. However, adopting this approach in robotics requires overcoming several barriers that often discourage the research community from following this promising avenue. The challenges range from the system integration of full-custom neuromorphic chips with sensors, conventional computing modules and motors, to the “programming” of the neural processing systems integrated on neuromorphic chips, up to the need for a principled framework for implementing and combining computational primitives, functions and operations in these devices using neural instead of digital representations.

Both conventional and neuromorphic robotics face the challenge of developing robust and adaptive modules to solve a wide range of tasks especially in applications in human-robot collaboration settings. Both will benefit from a framework designed to combine such modules to deliver a truly autonomous artificial agent. In this perspective, we discuss the current challenges of robotics and neuromorphic technology, and suggest possible research directions for overcoming current roadblocks and enabling the construction of intelligent robotic systems of the future, powered by neuromorphic technology.

## Requirements for intelligent robots

Recent developments in machine learning, supported by increasingly powerful and accessible computational resources, led to impressive results in robotics-specific applications^2–4^. Nevertheless, except for the case of precisely calibrated robots performing repetitive operations in controlled environments, autonomous operations in natural settings are still challenging due to the variability and unpredictability of the dynamic environments in which they act.

The interaction with uncontrolled environments and human collaborators requires the ability to continuously infer, predict and adapt to the state of the environment, of humans, and of the robotic platform itself, as described in Box 1. Current machine learning, deep networks, and AI methods for robotics are not best suited for these types of scenarios and their use still has critical roadblocks that hinder their full exploitation. These methods typically require high computational (and power) resources: for example deep networks have a very large number of parameters, they need to be trained with very large datasets, and require a large amount of training time, even when using large Graphics Processing Unit (GPU) clusters. The datasets used are mostly disembodied, while ideally, for robotic applications, they would need to be tailored^9^ and platform specific. This is especially true for end-to-end reinforcement learning, where the dataset depends on the robot plant and actuation. Data acquisition and dataset creation are expensive and time consuming. While virtual simulations can partially improve this aspect, transfer learning techniques do not always solve the problem of adapting pre-trained architectures to real-world applications. Off-line training on large datasets with thousands of parameters also implies the use of high performance, powerful but expensive and power-hungry computing infrastructures. Inference suffers less from this problem and can be run on less demanding, embedded platforms, but at the cost of very limited or no adaptation abilities, thus making the system brittle to real-world, ever-changing scenarios^10^.

The key requirements in robotics are hence to reduce or possibly eliminate the need for data- and computation-hungry algorithms, making efficient use of sensory data, and to develop solutions for continuous online learning where robots can acquire new knowledge by means of weak- or self-supervision. An important step toward this goal is moving from static (or frame-based) to dynamic (or event-based) computing paradigms, able to generalise and adapt to different application scenarios, users, robots, and goals.

Neuromorphic perception addresses these problems right from the sensory acquisition level. It uses novel bio-inspired sensors that efficiently encode sensory signals with asynchronous event-based strategies^11^. It also adopts computational primitives that extract information from the events obtained from the sensors, relying on a diverse set of spike-driven computing modules.

Neuromorphic behaviour follows control policies that adapt to different environmental and operating conditions by integrating multiple sensory inputs, using event-based computational primitives to accomplish a desired task.

Both neuromorphic perception and behaviour are based on computational primitives that are derived from models of neural circuits in biological brains and that are therefore very well suited for being implemented using mixed signal analogue/digital circuits^12^. This offers an efficient technological substrate for neuromorphic perception and actions in robotics. Examples are context-dependent cooperative and competitive information processing, and learning and adaptation at multiple temporal scales^13,14^.

**Fig. 1:**
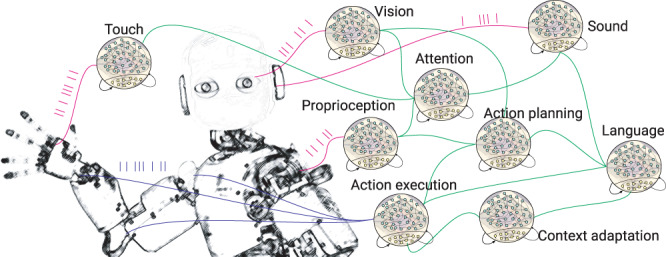
Robots with end-to-end neuromorphic intelligence. Some non exhaustive examples of perception (magenta), intelligent behaviour (green) up to action execution (blue) that would all be implemented by means of dedicated Spiking Neural Network (SNN) hardware technology. iCub picture ^©^IIT author Agnese Abrusci.

The development and integration of neuromorphic perception and behaviour using hardware neuromorphic computational primitives has the final goal of designing a robot with end-to-end neuromorphic intelligence as shown in Fig. 1.

In the next sections, we present an overview of the neuromorphic perception, action planning, and cognitive processing strategies, highlighting features and problems of the current state of the art in these domains. We conclude with a road map and a “call for action” to make progress in the field of embodied neuromorphic intelligence.

Box 1 The need for adaptation in robotics While the majority of industrial robots are currently operating in controlled settings to execute programmable and repetitive actions, robotics research is moving towards human-robot collaboration scenarios, where robots are expected to interact and collaborate with humans in uncontrolled environments in daily tasks^133,134^. Different individuals’ behavioural and environmental physical conditions might change across days and tasks. The ability of robots to adapt is hence crucial for functioning in the real world and interacting with humans^135^. In the majority of applications, including in industry, the robot plant wears out over time, and the controller needs to adapt to changes on the plant characteristics over very long time scales. In rehabilitation robotics, the controller needs to adapt to the progress of each individual during therapy as well as to different patients, requiring adaptation both over long and short temporal scales^136^. In most interactive applications robots must also be able to react to sudden environmental changes over short time scales, for example by switching to previously learned configurations. Unmanned autonomous robotic vehicles need to cope with changes in the environment, such as wind strength and direction; humanoid and roving robots need to adapt to different types of terrains^137^; artificial hands need to learn to manipulate objects of different weight and softness.

Biology provides a rich set of examples to address these needs, adapting to the changes described above^138,139^. On short time scales, biological systems can adapt away constant inputs with short-term plasticity mechanisms^140^; for longer time scales, their sensors can adapt their sensitivity to the level of the encoded signal (e.g., photoreceptors adapt to the global illumination level, to become more sensitive in dim illumination, or less sensitive under direct sun light)^141^. On very long time scales, homoeostatic mechanisms regulate the overall neural activity to keep it within defined bounds, thus coping with slow changes in the environment, or in the population’s overall drive^142^.

### Neuromorphic perception

Robots typically include many sensors that gather information about the external world, such as cameras, microphones, pressure sensors (for touch), lidars, time-of-flight sensors, temperature sensors, force-torque sensor,s or proximity sensors. In conventional setups, all sensors measure their corresponding physical signal and sample it at fixed temporal intervals, irrespective of the state and dynamics of the signal itself. They typically provide a series of static snapshots of the external world. When the signal is static, they keep on transmitting redundant data, but with no additional information, and can miss important samples when the signal changes rapidly, with a trade-off between sampling rate (for capturing dynamic signals) and data load. Conversely, in most neuromorphic sensory systems, the sensed signal is sampled and converted into digital pulses (or “events”, or “spikes”) only when there is a large enough change in the signal itself, using event-based time encoding schemes^15,16^ such as pulse-density or sigma-delta modulation^17^. The data acquisition is hence adapted to the signal dynamics, with the event rate increasing for rapidly changing stimuli and decreasing for slowly changing ones. This type of encoding does not lose information^18–20^ and is extremely effective in scenarios with sparse activity. This event-representation is key for efficient, fast, robust and highly-informative sensing. The technological improvement comprises a reduced need for data transmission, storage and processing, coupled with high temporal resolution – when needed – and low latency. This is extremely useful for real time robotic applications.

Starting from the design of motion sensors and transient imagers^21^, the first event-driven vision sensors with enough resolution, low noise and sensor mismatch – the Dynamic Vision Sensor (DVS)^22^ and Asynchronous Temporal Imaging Sensor (ATIS)^23^ – triggered the development of diverse algorithms for event-driven visual processing and their integration on robotic platforms^24^. These sensor information encoding methods break decades of static frame encoding as used by conventional cameras. Their novelty calls for the development of a new principled approach to event-driven perception. The event-driven implementation of machine vision approaches vastly outperforms conventional algorithmic solutions in specific tasks such as fast object tracking^25^, optical flow^26–28^ or stereo^29^ and Simultaneous Localisation and Mapping (SLAM)^30^. However, these algorithms and their hardware implementations still suffer from task specificity and limited adaptability.

**Fig. 2:**
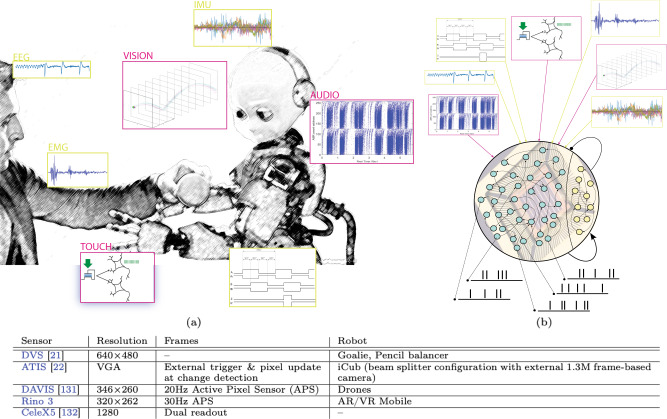
Neuromorphic sensing for robots. **a** the iCub robot (picture ^©^IIT author Duilio Farina) is a platform for integrating neuromorphic sensors. Magenta boxes show neuromorphic sensors that acquire continuous physical signals and encode them in spike trains (vision, audition, touch). All other sensors, that monitor the state of the robot and of its collaborators, rely on clocked acquisition (green boxes), that can be converted to spike encoding by means of Field Programmable Gate Arrays (FPGAs) or sub-threshold mixed-mode devices. **b** The output of event-driven sensors can be sent to Spiking Neural Networks (SNNs) (with learning and recurrent connections) for processing. VISION box in (**a**): Event-driven vision sensors produce “streams of events” (green for light to dark changes, magenta for dark to light changes). The trajectory of a bouncing ball can be observed continuously over space, with microsecond temporal resolution (black rectangles represent sampling of a 30 fps camera). Table: Event-driven vision sensors evolved from the Dynamic Vision Sensor (DVS) with only “change detecting” pixels - to higher resolution versions with absolute light intensity measurements. The Dynamic and Active pixel VIsion Sensor (DAVIS)^131^ acquires intensity frames at low frame rate simultaneously to the “change detection” (with minor cross talk and artefacts on the event stream during the frame trigger). The Asynchronous Temporal Imaging Sensor (ATIS)^132^ samples absolute light intensity only for those pixels that detect a change. The CeleX5 offers either frame-based or event-driven readout (with a few milliseconds delay between the two, resulting in loss of event stream data during a frame acquisition). Similar to the DAVIS, the Rino3 captures events and intensity frames simultaneously, however, it employs a synchronised readout architecture as opposed to the asynchronous readout typically found in other event-driven sensors. The ultimate solution combining frames and events is yet to be found. Merging two stand-alone sensors in a single optical setup poses severe challenges in terms of the development of optics that trade-off luminosity with bulkiness. Merging two types of acquisition on the same sensor limits the fill-in factor and increases noise and interference between frames and events.

**Fig. 3:**
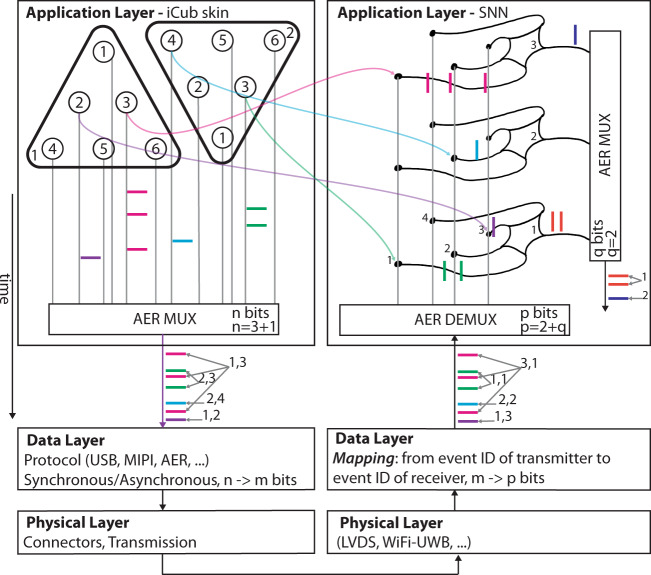
AER: example of communication between an event-driven sensor (triangular skin patches, each with 6 sensing areas) and a spiking neural network (SNN) chip. Each sensing element emits asynchronous spikes that are sent to a bus through arbitration. The same are de-multiplexed to be sent to the correct synapse of the SNN chip.

These event-driven sensory-processing modules will progressively substitute their frame-based counterparts in robotic pipelines (see Fig. 2). However, despite the promising results, the uptake of event-driven sensing in robotics is still difficult due to the mindset change that is required to work with streams of events, instead of static frames. Furthermore, this new data representation calls for the development of new ad hoc interfaces, communication protocols (described in Box 2 and Fig. 3) and software libraries for handling events. Open source JAVA^31^ and C++^32,33^ libraries have already been developed, also within two of the main robotic middlewares – ROS and YARP – but they require additional contributions from a large community to grow and reach the maturity needed for successful adoption in robotics. Eventually, a hybrid approach that combines frame-based and event-driven modules, and that fosters the growth of the community revolving around it, could favour a more widespread adoption in the robotics domain. However, this hybrid neuromorphic/traditional design strategy would not fully exploit all the advantages of the neuromorphic paradigm.

Working towards the implementation of robots with full neuromorphic vision, the neuromorphic and computational neuroscience communities have started in-depth work on perceptive modules for stereo vision^34^ and vergence^35^, attention^36^, and object recognition^37^. These algorithms can run on neuromorphic computing substrates for exploiting efficiency, adaptability and low latency.

The roadmap of neuromorphic sensor development started with vision, loosely inspired by biological photo-transduction, and audition, inspired by the cochlea, and only later progressed to touch and olfaction. The event-driven acquisition principle is extremely valuable also when applied to other sensory modalities, especially those characterised by temporally and spatially localised activation, such as tactile, auditory, and force-torque modalities, those requiring extremely low-latency for closed-loop control, such as encoders and Inertia Measurement Units (IMUs), non-biological like sensors that augment the ability to monitor the environment, such as lidar, time-of-flight, 3D, and proximity sensors, and sensors that help the robot to monitor the state of human beings, e.g. Electromyography (EMG), Electroencephalography (EEG), centre of mass, etc.^38^.

Available cochlear implementations rely either on sub-threshold mixed-mode silicon devices^39,40^ (as do the vision sensors), or on Field Programmable Gate Arrays (FPGAs)^41^. They have been applied mostly to sound source localisation and auditory attention, based on the extremely precise temporal footprint of left and right signals^42,43^, and, lately, on audio-visual speech recognition^44^. Their integration on robots, however, is still very limited: as in event-driven vision, they require application development tools, and a way in which they can be exploited in speech processing.

The problem of tactile perception is further complicated by three factors. First, by the sheer number of available different physical transducers. Second, by the difficulty in interfacing the transducers to silicon readout devices. This is unlike the situation in vision, where silicon photo-diodes can capture light and are physically part of the readout device. Third, there are the engineering challenges in integrating tactile sensors on robotic platforms, comprising miniaturisation, and design and implementation on flexible and durable materials with good mechanical properties, wiring, and robustness. Very few native neuromorphic tactile sensors have been developed so far^45–48^ and none has been stably integrated as part of a robotic platform, besides lab prototypes. While waiting for these sensors to be integrated on robots, existing integrated clock-based sensing can be used to support the development of event-driven robotics applications. In this “soft” neuromorphic approach, the front end clocked samples are converted to event-based representation by means of algorithms implemented in software^49–51^ or embedded on Digital Signal Processors (DSPs)^52^ or FPGAs^53,54^. The same approach is valuable also in other sensory modalities, such as proprioception^55,56^, to support the development of event-driven algorithms and validate their use in robotic applications. However, it is not optimal in terms of size, power, and latency.

For all sensory modalities, the underlying neuromorphic principle is that of “change detection”, a high level abstraction that captures the essence of biological sensory encoding. It is also a well defined operation that allows algorithms and methods to extract information from data streams^15^ to be formalised. Better understanding the sophisticated neural encoding of the properties of the sensed signal and their relation to behavioural decisions of the subject^57^ – and their implementation in the design of novel neuromorphic sensors – would enhance the capability of artificial agents to extract relevant information and take appropriate decisions.

Box 2 Neuromorphic communication protocols Like neural systems, neuromorphic systems rely on digital communication: information is encoded in the timing of voltage pulses (or spikes). Biological neurons have dedicated connections with huge fan-in and fan-out, supported by the three-dimensional structure of the neural tissue. Silicon neurons instead can only use wires on two-dimensional planes, but they can exploit the speed of metal wires that are orders of magnitude faster than axons. These limits in physical connectivity can therefore be partially solved by adopting temporal multiplexing techniques that use the same physical wires to send spikes of different neurons. To distinguish the spikes that travel on the same wire, the identity of the source or destination neuron is encoded with a digital world, implementing what is known as the Address Event Representation (AER) protocol^143^.

AER has been implemented by the neuromorphic community since the late 90’s^144–146^, in many different setups and variants. The need for integrating this communication protocol on robotic platforms defines a set of requirements such as sparsity of event-communication, high noise rejection, low-latency, sufficient bandwidth, and a minimum number of wires that can lead to the definition of a widely adopted standard. In robotic applications that combine multiple distributed sensors, asynchronous serial implementations are preferable^147^, as the use of synchronous protocols would require including and synchronising multiple clocks. Given the recent uptake of neuromorphic technologies by large industries and the growth of the research community, the definition of a common standard is necessary and timely, to allow interoperability across different sensing, computing and actuating modules. The communication protocol can be standardised and optimised following the definition of application, data and physical layers of Fig. 3. The application layer comprises a neuromorphic component that sends or receives asynchronous address events. At this level, time represents itself: events are communicated asynchronously at the time in which they occur. Events are bundled together into larger packets with either fixed or varying sizes in the data layer. This is a required step, if well established standards such as MIPI or USB are also going to be used. Interfacing AER to synchronous implementations requires to embed the precise timing information of the events within the data stream (e.g., by time-stamping). The physical layer defines the means of transmitting the actual bits. To accommodate the bandwidth required by state-of-the-art vision sensors, well-established high-speed communication standards, such as differential signalling may be used. For each layer the community will have to define common specifications, and develop the necessary interfacing circuits for on-chip integration, removing the need for bridging devices such as Field Programmable Gate Arrays (FPGAs). In this perspective, the definition of a standard application layer would decrease the cost of the development of a number of application specific interfaces. However, the definition of requirements for the optimal protocol is still an open question in the community and strongly depends on the application.

### Neuromorphic behaviour

To interact efficiently with the environment, robots need to choose the most appropriate behaviour, relying on attention, allocation, anticipation, reasoning about other agents, planning the correct sequence of actions and movements based on their understanding of the external world and of their own state. Biological intelligent behaviour couples the ability to perform such high level tasks with the estimation, from experience, of the consequences of future events for generating goal-oriented actions.

A hypothesis for how intelligent behaviour is carried out by the mammalian nervous system is the existence of a finite set of computational primitives used throughout the cerebral cortex. Computational primitives are building blocks that can be assembled to extract information from multiple sensory modalities and coordinate a complex set of motor actions that depend on the goal of the agent and on the contingent scenario (e.g. presence of obstacles, human collaborators, tools).

The choice of the most appropriate behaviour, or action, in the neuromorphic domain is currently limited to proof-of-concept models. Box 3 reviews the state-of-the-art of robots with sensing and processing implemented on neuromorphic devices. Most implementations consist of a single bi-stable network discriminating between ambiguous external stimuli^58^ and selecting one of two possible actions. Dynamic Field Theory (DFT) is the reference framework for modelling such networks, where the basic computational element is a Dynamic Neural Field (DNF)^59^, computationally equivalent to a soft Winner-Take-All (WTA). As described in Box 4, WTA networks are one of the core computational primitives that can be implemented in neuromorphic hardware. Therefore, DNF represents an ideal framework which can translate intelligent models into feasible implementations in a language compatible with neuromorphic architectures^60^. The current challenge in such systems is to develop a multi-area and multi-task spiking neuron model of the cortical areas involved in decision making under uncertainty.

Different branches of robotics have tackled this challenge by exploring biologically inspired embodied brain architectures to implement higher-level functions^61^ to provide robots with skills to interact with the real world in real-time. These architectures are required to learn sensorimotor skills through interaction with their environment and via incremental developmental stages^62,63^.

Once the appropriate behaviour is selected, it has to be translated into a combination of actions, or dynamic motor primitives, to generate rich sets of complex movements and switching behaviours, for example switching between different rhythmic motions such as walking, generated via a Central Pattern Generator (CPG), and swimming^64^. The stability and capability of these systems in generating diverse actions is formally proven^65^. This motivates their adoption and further progress to biological plausibility with spiking implementations^66^. As a result, robots benefit from the biology of animal locomotor skills and can be used as tools for testing animal locomotion and motor control models and how they are affected by sensory feedback^67^.

Despite taking its inspiration from neural computation, robotics inspired by neural systems has only recently started to use Spiking Neural Networks (SNNs) and biologically plausible sensory input, and the corresponding computational substrate that can support SNNs and learning. Neuromorphic technologies move one step further in this direction. In recent years there has been substantial progress in developing large-scale brain inspired computing technologies^68–71^ that allow the exploration of the computational role of different neural processing primitives to build intelligent systems^72–74^. Although knowledge of the neural activity underlying those functions is increasing, we are not yet able to explicitly and quantitatively connect intelligence to neural architectures and activity. This hinders the configuration of large systems to achieve effective behaviour and action planning. An example of an attempt to develop tools to use spiking neurons as a basis to implement mathematical functions is the “Neural Engineering Framework (NEF)”^75^, that has been successfully deployed to implement adaptive motor control for a robotics arm^76^. The NEF formalisation allows the use of neurons as computational units, implementing standard control theory, but overlooks the brain architectures and canonical circuits that implement the same functionalities.

Current research on motor control implementation based on brain computational primitives mainly focuses on the translation of well-established robotic controllers into SNNs that run on neuromorphic devices^56,77–79^. Although the results show the potential of this technology, these implementations still need to follow a hybrid approach in which neuromorphic modules have to be interfaced to standard robotics ones. In the example cited above, motors are driven via embedded controllers with proprietary algorithms and closed/inaccessible electronic components. There is therefore the need to perform spike encoding of continuous sensory signals measured by classical sensors, and to perform decoding from spike trains to signals compatible with classical motor controllers. This inherently limits the performance of hybrid systems that would benefit from being end-to-end event-based. In this respect, the performance of the standard motor controller and its spiking counterpart cannot be benchmarked on the same robotic task, because of the system-level interfacing issues. To make inroads toward the design of fully neuromorphic end-to-end robotic systems, it is essential to design new event-based sensors (e.g. IMU, encoders, pressure) to complement the ones already available (e.g. audio, video, touch). In addition, motors or actuators should be directly controlled by spike trains, moving from Pulse Width Modulation (PWM) to Pulse Frequency Modulation (PFM)^80–82^. Furthermore, the end-to-end neuromorphic robotic system could benefit from substituting the current basic methods used in robotics (e.g. Model Predictive Control (MPC), Proportional Integral Derivative (PID)) with more biologically plausible ones (e.g. motorneuron – Golgi – muscle spindle architectures^83^) that can be directly implemented by the spiking neural network circuits present on neuromorphic processors. The drawback of this approach, however, lies in the limited resolution and noisy computing substrate used in these processors, as well as in the lack of an established control theory that uses the linear and non-linear operators present in spiking neural networks (e.g. integration, adaptation, rectification). The proposed biologically inspired control strategies would probably benefit from the use of bio-inspired actuators, such as tendons^48^, agonist-antagonist muscles^84^, soft actuators^85^. While offering more compliant behaviour, these introduce non-linearities that are harder to control with traditional approaches, but match the intrinsic properties of biological actuation, driven by networks of neurons and synapses.

Box 3 Neuromorphic robots **Wheeled robots** Wheeled robots are often used to implement spatial navigation tasks. However, despite recent advances in research^30,148–150^, robots are still not able to compete with biological systems in terms of robustness to the changes in the visual scene for map formation, or in terms of power and resource efficient ways to store maps and path-planning data. Neuromorphic wheeled robots are being used to validate studies of how the nervous system accomplishes these tasks with low power and limited resources (e.g. by using spiking neural networks). These studies are still in the early stages, however successful examples already exist of basic navigation tasks (such as turning left/right or tuning the robot’s speed) implemented using hardware Spiking Neural Networks (SNNs) in small robotic agents^58,148,151,152^.

**iCub** The iCub is a humanoid robot that can be used to perform closed-loop experiments with neuromorphic devices, since it supports the use of event-driven vision and touch sensors that can be interfaced to neuromorphic processors. In ref. ^56^ the authors present a neuromorphic architecture for head pose estimation and scene representation realised using the Loihi neuromorphic processor^70^. The network integrates motor commands to estimate the iCub’s head pose in a neural path-integration process based on Dynamic Neural Field (DNF). In ref. ^55^ a closed-loop PID controller was implemented using relational neural networks to control the iCub’s head rotation. The network was implemented using the mixed-signal DYNAP-SE neuromorphic processor^69^. In ref. ^153^ the Vestibulo-Ocular Reflex (VOR) was implemented using a spiking cerebellar model within an adaptive real-time control loop. The VOR protocols moved the iCub head and the eyes which incorporate a camera that can be used to check the image motion on the “retinas”. In these proof-of-concept, the robot shows adaptation behaviour, however, limited in one DoF.

**Drones** SNNs represent a promising tool for controlling resource-constrained agents that require fast reaction times, such as Unmanned Aerial Vehicles (UAVs) thanks to their low-latency and fast response times. In ref. ^154^ a drone was able to perform optic flow landings with an evolved SNN running at high frequencies (over 250 kHz). The performance compared to conventional mobile GPU shows 75 × lower power, without any loss in performance, but again for a single DoF. A similar work interfaced Loihi to a UAV to control a single DoF using a spiking Proportional Integral Derivative (PID). The controller is built using neuronal populations, in which single spikes carry information about sensory and control signals^77^.

**Robotic arms** In ref. ^155^ the authors compared two different platforms, Loihi and SpiNNaker2 on a common benchmark, the control of a robotic arm, in terms of computation time and active energy. Both platforms are efficient in specific parameter regions, SpiNNaker2 is more efficient when the number of input dimensions is high, while Loihi is more efficient when the number of input dimensions is low. Another example deploys Neural Engineering Framework (NEF)-based neuromorphic algorithms for inverse kinematics and a PID for the control of a six-DoF robotic arm^156^. The algorithms are designed using Nengo and evaluated on Loihi. Similarly, in ref. ^79^ a spiking PID is used to control a four-DoF robotic arm. Combining the spiking PID with PFM motor control, the system achieves a current consumption below 1A when all the motors are working at the same time. The controller is implemented on an Field Programmable Gate Array (FPGA), and the robot joints’ commands can be received from a population of silicon-neurons running on the DYNAP-SE platform that generates the required Pulse Frequency Modulation (PFM) reference signals for the FPGA.

**Legged-robot** Central Pattern Generator (CPG) is a computational primitive that generates and controls rhythmic movements. Spiking CPG are used in insect robots’ locomotion, to coordinate single leg movements and the coordination of multiple legs. Spiking CPG show stable and coordinated locomotion pattern that can robustly adapt to external disturbances^157^ and can be implemented on FPGA^158^.

Box 4 A dictionary of hardware neural primitives **Sensors** transduce analogue and continuous physical signals into electrical discrete pulses that emulate neural sensory encoding. Depending on the physical position, shape and local computation, they can pre-process the sensory signal in non-trivial ways. For example, in vision, neuromorphic sensors work as edge extractors^11^, neuromorphic cochleae act as frequency tuned filters^159^.

**Neurons** integrate information from different sources over time and, depending on multiple factors that influence their state, communicate the result of a non-trivial analogue computation to other neurons by means of digital voltage pulses (action potentials, or spikes). Starting from the silicon implementation of the Hodgkin and Huxley neuron model^160^, in which various ion currents modulate the membrane potential^161^, more compact circuits have been proposed to improve the trade-off between accurate modelling and functional behaviour. The Leaky Integrate-and-Fire (LIF)^162^ model captures the principle of integrating spikes over time and producing an output firing activity proportional to the input. Generalised LIF circuits reproduce neurons’ characteristic bursting behaviours^163–165^.

**Synapses** connect neurons and mediate the propagation of information between neurons. Their most simple implementation is a switch that injects a fixed amount of current into the membrane of neurons; more faithful implementations use a handful of transistors to add the temporal dynamics of the post-synaptic current^166^. The information is transmitted through excitatory or inhibitory connections, to increase or decrease the activity in the receiving neuron.

**Plasticity** is the mechanism that modifies the behaviour of neural computation and synaptic transmission depending on the state of the synapses and the input activity. It supports adaptation and learning. A number of circuits implement short-time (in the order of tens of milliseconds) activity-dependent plasticity, such as Short-Term Depression (STD)^167^ and Short-Term Facilitation (STF)^168^, or Spike Frequency Adaptation (SFA)^169^, useful to enhance changes in the transmitted information and filter constant activity. Long-term (in the order of seconds) plasticity driven by the coincident activation of connected neurons supports Hebbian types of learning^170–175^. Progress in nanoscale technologies^46,176–178^ is contributing to the dictionary of hardware plasticity primitives, towards dense integration. Within long-term plasticity, multiple temporal scales in the learning synapses increase the memory capacity of networks using discrete and bound states^179^. Very long-term plasticity (in the order of days) supports homoeostatic regulation of the overall network activity. This is kept within functional ranges in the face of long-term modifications of the network or changes in the input stimuli^14^.

**Neural oscillators** are found in neural cortex and rely on two mutually connected neural populations to support feature binding and motor coordination through the generation of rhythmic activity. A specific instance of neural oscillators are Central Pattern Generator (CPG). These rely on neurons SFA and are capable of generating a rich set of complex movements and switching behaviours supporting walking, swimming, and flying^100^.

**Delay/Temporal measurement** circuits take inspiration from the insect brain, where motion is computed as the time to travel of a stimulus from one sensing element to the neighbour^180^. This type of computational primitive is useful for motion estimation and obstacle avoidance^88^.

**Cooperative-competitive networks** rely on networks of neurons that are recurrently connected. Functionally, they process information in a way that takes into account the context and the relative activation of different units. Recurrent inhibition to an excitatory population helps improving the selectivity of neurons to a specific feature, as neurons with similar selectivity reinforce each other’s response and inhibit the response of other neurons that are tuned to different features^34,181^. Relational networks use recurrent connectivity to express relative dependencies between variables, for example to compute the error between a measured signal and its target value^78^.

**Actuators** move and control parts of the body, to achieve a desired action. Different types of actuators exist in robotics that rely on different physical properties.

### Computational primitives for intelligent perception and behaviour

In addition to the adoption of neuromorphic sensors, the implementation of fully end-to-end neuromorphic sensorimotor systems requires fundamental changes in the way signals are processed and computation is carried out. In particular, it requires replacing the processing that is typically done using standard computing platforms, such as microcontrollers, DSPs, or FPGA devices, with computational primitives that can be implemented using neuromorphic processing systems. That is to say, computational primitives implemented by populations of spiking neurons that act on the signals obtained from both internal and external sensors, that learn to predict their statistics, that process and transform the continuous streams of sensory inputs into discrete symbols, and that represent internal states and goals. By supporting these computational primitives in the neuromorphic hardware substrate, such an architecture would be capable of carrying out sensing, planning and prediction. It would be able to produce state-dependent decisions and motor commands to drive robots and generate autonomous behaviour. This approach would allow the integration of multiple neuromorphic sensory-processing systems distributed and embedded in the robot body, closing the loop between sensing and action in real-time, with adaptive, low-latency, and low power consumption features.

Realising a hardware substrate that emulates the physics or biological neural processing systems and using it to implement these computational primitives can be considered as a way to implement embodied intelligence. In this respect one could consider these hardware computational primitives as “elements of cognition”^86^, that could bridge the research done on embodied neuromorphic intelligence with that of cognitive robotics^87^.

Several examples of neuromorphic processing systems that support the implementation of brain-inspired computational primitives by emulating the dynamics of real neurons for signal processing and computation have already been proposed^42,69,88^. Rather than using serial, bit-precise, clocked, time-multiplexed representations, these systems make use of massively parallel in-memory computing analogue circuits. Recently, there has also been substantial progress in developing large-scale brain-inspired computing technologies that follow this parallel in-memory computing strategy, in which silicon circuits can be slowed down to the time-scales relevant for robotic applications^69,71,89^. By implementing computational primitives through the dynamics of multiple parallel arrays of neuromorphic analogue circuits, it is possible to bypass the need to use clocked, time-multiplexed circuits that decouple physical time from processing time, and to avoid the infamous von Neumann bottleneck problem^7,8,90^, which requires to shuffle data back and forth at very high clock-rates from external memory to the time-multiplexed processing unit. Although the neuromorphic approach significantly reduces power consumption, it requires circuits and processing elements that can integrate information over temporal scales that are well matched to those of the signals that are being sensed. For example, the control of robotic joint movements, the sensing of voice commands, or tracking of visual targets or human gestures would require the synapse and neural circuits to have time constants in the range of 5 ms to 500 ms. In addition to the technological challenge of implementing compact and reliable circuit elements that can have such long-lasting memory traces, there is an important theoretical challenge for understanding how to use such non-linear dynamical systems to carry out desired state-dependent computations. Unlike conventional computing approaches, the equivalent of a “compiler” tool that allows the mapping of a desired complex computation or behaviour into a “machine-code”-level configuration of basic computing units such as dynamic synapses or Integrate-and-Fire neurons is still lacking. One way to tackle this challenge, is to identify a set of brain-inspired neural primitives that are compatible with the features and limitations of the neuromorphic circuits used to implement them^12,91–94^ and that can be combined and composed in a modular way to achieve the desired high-level computational primitive functionality. Box 4 lists a proposed dictionary of such primitives.

In addition, the computational requirements of robotic systems have to treat also sensors and actuators as computational primitives that shape the encoding of the sensory signal and of the movements depending on their physical shape (e.g. composite eyes, versus retina-like foveated or uniform vision sensors, brushless and DC-motors versus soft actuators), location (e.g. binocular versus monocular vision, non-uniform distribution of tactile sensors and location of the motor with respect to the body part that has to be moved) and local computation (e.g. feature extraction in sensors or low-level closed-loop control).

Based on the required outcome, neural circuits can be endowed with additional properties that implement useful non-linearities, such as Spike Frequency Adaptation (SFA) or refractory period settings. These building blocks can be further combined to produce computational primitives such as soft WTA networks^95–99^, neural oscillators^100^, or state-dependent computing networks^7,12,101^, to recognise or generate sequences of actions^8,78,102–107^. By combining these  with sensing and actuation neural primitives, they can produce rich behaviour useful in robotics.

### WTA networks

WTA networks represent a common “canonical” circuit motive, found throughout multiple parts of the neocortex^108,109^. Theoretical studies have shown that such networks provide elementary units of computation that can stabilise and de-noise the neuronal dynamics^108,110,111^. These features have been validated with neuromorphic SNN implementations to generate robust behaviour in closed sensorimotor loops^97,101,112–114^. WTA networks composed of *n* units can be used to represent *n* valued variables, with population coding. In this way it is possible to couple multiple WTA networks among each other and implement networks of relations among different variables^115,116^ (e.g. to represent the relationship between a given motor command value and the desired joint angle^78^). As WTA networks can create sustained activation to keep a neuronal state active even after the input to the network is removed, they provide a model of working memory^100,102,117,118^. WTA dynamics create stable attractors are computationally equivalent to DNF that enable behaviour learning in a closed sensorimotor loop in which the sensory input changes continually as the agent generates action. In order to learn a mapping between a sensory state and its consequences, or a precondition and an action, the sensory state before the action needs to be stored in a neuronal representation. This can be achieved by creating a reverberating activation in a neuronal population that can be sustained for the duration of the action even if the initial input ceases. The sustained activity can be used to update sensorimotor mappings when a rewarding or punishing signal is obtained^60,119^. Finally, these attractor-based representations can bridge the neuron circuit dynamics with the robot behavioural time scales in a robust way^8,118,120^, and be exploited to develop more complex embedded neuromorphic intelligent systems. However, to reach this goal, it is necessary to develop higher-level control strategies and theoretical frameworks that are compatible with mixed signal neuromorphic hardware, which have compositionality and modularity properties.

### State-dependent intelligent processing

State-dependent intelligent processing is a computational framework that can support the development of more complex neuromorphic intelligent systems. In biology, real neural networks perform state-dependent computations using WTA-type working memory structures maintained by recurrent excitation and modulated by feedback inhibition^121–126^. Specifically, modelling studies of state-dependent processing in cortical networks have shown how coupled WTA networks can reproduce the computational properties of Finite State Machines (FSMs)^101,123,127^. An FSM is an abstract computing machine that can be in only one of its *n* possible states, and that can transition between states upon receiving an appropriate external input. True FSMs can be robustly implemented in digital computers that can rely on bit-precise encoding. However, their corresponding neural implementations built using neuromorphic SNN architectures, are affected by noise and variability, very much like their biological counterparts. In addition to exploiting the stabilising properties of WTA networks, the solution that neuromorphic engineers found to implement robust and reliable FSM state-dependent processing with noisy silicon neuron circuits is to resort to dis-inhibition mechanisms analogous to the ones found in many brain areas^128,129^. These hardware state-dependent processing SNNs have been denoted as Neural State Machines (NSMs)^101,105^. They represent a primitive structure for implementing state-dependent and context-dependent computation in spiking neural networks. Multiple NSMs can interact with each other in a modular way and can be used as building blocks to construct complex cognitive computations in neuromorphic agents^105,130^.

Neuromorphic sensors, computational substrates and actuators are combined to build autonomous agents endowed with embodied intelligence, by means of brain-like asynchronous, digital communication. Existing agents range from monolithic implementations - whereby sensor is directly connected to a neuromorphic computing device - to modular implementations, where distributed sensors and processing devices are connected by means of a middleware abstraction layer, trading off compactness and task-specific implementations with flexibility. Both approaches would benefit from the standardisation of the communication protocol (discussed in Box 2).

## Outlook

Embodied neuromorphic intelligent agents are on their way. They promise to interact more smoothly with the environment and with humans by incorporating brain-inspired computing methods. They are being designed to take autonomous decisions and execute corresponding actions in a way that takes into account many different sources of information, reducing uncertainty and ambiguity from perception, and continuously learning and adapting to changing conditions.

In general, the overall system design of traditional robotics and even current neuromorphic approaches is still far from any biological inspiration. A real breakthrough in the field will happen if the whole system design is based on biological computational principles, with a tight interplay between the estimation of the surroundings and the robot’s own state, and decision making, planning and action. Scaling to more complex tasks is still an open challenge and requires further development of perception and behaviour, and further co-design of computational primitives that can be naturally mapped onto neuromorphic computing platforms and supported by the physics of its electronic components. At the system level, there is still a lack of understanding on how to integrate all sensing and computing components in a coherent system that forms a stable perception useful for behaviour. Additionally, the field is lacking a notion of how to exploit the intricate non-linear properties of biological neural processing systems, for example to integrate adaptation and learning at different temporal scales. This is both on the theory/algorithmic level and on the hardware level, where novel technologies could be exploited, for such requirements.

The roadmap towards the success of neuromorphic intelligent agents encompasses the growth of the neuromorphic community with a cross-fertilisation with other research communities, as discussed in Box 5, Box 6.

The characteristics of neuromorphic computing technology so far have been demonstrated by proof of concept applications. It nevertheless holds the promise to enable the construction of power-efficient and compact intelligent robotic systems, capable of perceiving, acting, and learning in challenging real-world environments. A number of issues need to be addressed before this technology is mature to solve complex robotic tasks and can enter mainstream robotics. In the short term, it will be imperative to develop user-friendly tools for the integration and programming of neuromorphic devices to enable a large community of users and the adoption of the neuromorphic approach by roboticists. The path to follow can be similar to the one adopted by robotics, with open source platforms and development of user-friendly middleware. Similarly, the community should rely on a common set of guiding principles for the development of intelligence using neural primitives. New information and signal processing theories should be developed following these principles also for the design of asynchronous, event-based processing in neuromorphic hardware and neuronal encoding circuits. This should be done with the cross-fertilisation of the neuromorphic community with computational neuroscience and information theory; furthermore interaction with materials and (soft-)robotics communities will better define the application domain and the specific problems for which neuromorphic approaches can make a difference. Eventually, the application of a neuromorphic approach to robotics will find solutions that are applicable in other domains, such as smart spaces, automotive, prosthetics, rehabilitation, and brain-machine interfaces, where different types of signals may need to be interpreted, to make behavioural decisions and generate actions in real-time.

Box 5 Call for actions **Call for the neuromorphic community** To favour the uptake and the building of a larger community of users and stakeholders of embodied neuromorphic intelligence, the neuromorphic community should focus on the design of modular and reusable sensing and computing modules. The standardisation of a common communication protocol, as described in Box 2, has already enabled sharing of modules and systems. Open-source implementations of algorithms and dataset-sharing will promote the growth of the field. A milestone on this path will be the definition of a suite of benchmarks that can be used to quantitatively compare the features and benefits of different neuromorphic systems, as described in Box 6.

**Call for the computational neuroscience community** Neuromorphic circuits need to convert sensory signals into address-events for further processing. The computational neuroscience community has a unique opportunity to inspire and educate neuromorphic engineers by pointing out the principles and strategies that the nervous system uses to convert analogue inputs to spikes and encode sensory signals. Tight collaboration with the neuroscience community will lead to important improvements in neuromorphic sensing circuits^57,182^. Similarly, this community can provide useful insights for designing recurrent Spiking Neural Networks (SNNs) composed of noisy and inhomogeneous circuits to carry out signal processing and computation^183–185^. In this respect, it will be important to link specific neuroscience observations to their most basic computational role in order to isolate the basic mechanisms that are sufficient to implement a given functionality. The hardware implementation will then reproduce such a reduced “minimalist” model, where features, complexity, detail, and diversity have corresponding computational functions.

**Call for the material science community** Emerging memory technologies hold great promises for improving conventional computing architectures, However, they also represent an important opportunity for designing new types of solid-state nano-scale devices that could directly emulate the physics of real synapses, and therefore provide the computing substrate for implementing the principles of neural computation more efficiently. The material science community should therefore attempt to embrace and exploit the non-linear physics of these devices to optimise the design of embodied neuromorphic computing architectures^94^.

**Call for the computer science community** Similar to how computers use a hierarchy of levels of abstraction to manage the definition of complex operations, computer science can leverage on the notions and tools developed so far to define new methods for combining neural computational primitives, as those described in Box 4, to achieve intelligent functionalities^186^. A challenge that lays ahead is also how to formalise computation using non-linear dynamics, stochastic, and probabilistic methods, including embodiment in the robotic platform.

**Call for the soft robotics community** As the neuromorphic approach is a good fit for complex systems where the control is non-trivial, it is a perfect match to soft robotics. There is a need for undefined, providing use cases to the neuromorphic community. The resulting perceptive and cognitive functions – implemented using neuromorphic computational substrates – must be embedded on robots, where the morphology of the platform can influence the way sensory signals are acquired (e.g. through a different placement of the sensors) and the way actions are executed (e.g. different kinds of locomotion, rigid versus soft actuation, etc.). Neuromorphic engineering, thanks to its ability to implement adaptive circuits and systems for solving non-linear control systems, can offer a solution to the complex control of soft robots.

Box 6 Data sets and benchmarks The definition of benchmark tasks and data sets that are appropriate for evaluating the performance of the different neuromorphic processors and behaving systems is a difficult and challenging endeavour that has not been fully solved yet^187^. While most existing datasets, developed mainly by the machine learning community, rely on large collections of static data, neuromorphic datasets should take into account the different spatial and temporal representations used by neuromorphic systems. There have been indeed attempts at creating novel datasets useful for benchmarking event-based processing algorithms and methods^188–192^. However, these data sets are useful only for comparing a very limited set of systems and approaches. Specific benchmarks for evaluating spatio-temporal abilities of neuromorphic systems will need to go beyond the standard figures of merit from machine learning. To validate and compare the vast spectrum of brain-inspired neuromorphic behaving systems it will be necessary to define multiple sets of benchmarks that can be used to evaluate the performance of the system from end-to-end, for complex tasks. Examples of computations that should be evaluated include spatio-temporal pattern recognition, prediction, attention, decision making, memory, language, and spatial perception, as well as regression, clustering, and dimensionality reduction. Taken individually, these tasks are common to some of the problems being tackled by the machine learning community. But the neuromorphic systems should include also how the performance changes as a function of the resources used. Unlike machine learning, neuromorphic systems are designed to minimise memory and power consumption. So the benchmark figures of merit should include also the savings in power consumption (e.g. for autonomous robots), the reduction in volume and weight (e.g. for drones), the reduction in latency and response time, the maximisation of robustness to noise and changes in both the input signals and system’s internal state. Memory and time are also important dimensions to consider for these benchmarks. Given that neuromorphic systems use “in-memory computing” and do not have access to external memory banks for accessing information at arbitrary times, benchmarks need to evaluate how well neuromorphic systems can operate in tasks in which the system is required to associate signals that are being perceived in the present with data that was measured seconds, minutes, or even hours before. The development of appropriate tasks to assess the memory performance of neuromorphic systems for appropriately producing the desired behaviour is a challenge in itself. Once the task is defined, the benchmark will need to take into account also the other robustness, latency, or power figures of merit discussed above. Standard figures of merit currently used to evaluate conventional processors and computing systems, such as accuracy, floating point operation per second (FLOPS), tera operations per second (TOPS) or multiply and accumulate (MAC) operations per second are not appropriate in this case. It will be important to converge on a set of figures of merit that can be used and accepted by the neuromorphic community at large.
