# Author Correction: Embodied neuromorphic intelligence

**DOI:** 10.1038/s41467-022-29119-5

**Published:** 2022-03-11

**Authors:** Chiara Bartolozzi, Giacomo Indiveri, Elisa Donati

**Affiliations:** 1 Event-Driven Perception for Robotics, Istituto Italiano di Tecnologia, via San Quirico 19D, 16163 Genova, Italy; 2 Institute of Neuroinformatics, University of Zurich and ETH Zurich, Winterthurerstr. 190, 8057 Zurich, Switzerland

**Keywords:** Electrical and electronic engineering, Applied mathematics, Computational science, Computer science, Electronic devices

Correction to: *Nature Communications* 10.1038/s41467-022-28487-2, published online 23 February 2022.

The original version of this Article contained an error in the Reference section (Ref. 46), which was previously incorrectly given as

‘Neuromorphic tactile system encompassing healable materials and memristive elements to perform proof-of-concept edge tactile sensing, demonstrated in a prosthetic application.’

The correct version states

‘Neuromorphic tactile system encompassing healable materials and memristive elements to perform proof-of-concept edge tactile sensing, demonstrated in a robotic task that is further applicable to prosthetic applications.’

This has been corrected in both the PDF and HTML versions of the Article.

